# Efficacy of Neuro-muscular Electrical Stimulation for Orthostatic Hypotension Associated with Long-term Disuse and Diabetic Autonomic Neuropathy: A Case Report

**DOI:** 10.1298/ptr.E10298

**Published:** 2024-08-27

**Authors:** Kazuyuki KOMINAMI, Masatoshi AKINO, Motoshi KANAI

**Affiliations:** 1 Department of Rehabilitation, Sanseikai Kitano Hospital, Japan; 2 Department of Internal Medicine, Sapporo Kiyota Hospital, Japan; 3 Department of Internal Medicine, Sanseikai Kitano Hospital, Japan

**Keywords:** Orthostatic hypotension, Blood pressure, Neuromuscular electrical stimulation, Diabetic autonomic neuropathy, Physical therapy

## Abstract

Patient Background: A 75-year-old man had difficulty moving around at home because of loss of appetite and neglect of medication for several days. He was brought to the emergency room and admitted on the same day with a diagnosis of dehydration and diabetic ketoacidosis. He started physical therapy (PT), had frequent fainting and presyncope due to hypotension, and had difficulty leaving bed. The patient was transferred to our hospital to continue PT. Test results on admission were as follows: short physical performance battery (SPPB) [points], 1/12 points; chair stand 5 times (CS5) [sec], not possible; functional independent measure (FIM) [points], 66/126; standing test: blood pressure (BP) [mmHg], 130/60/HR [beats per minute], 76 in supine, 90/56/79 in sitting, 70/–/79 in standing. Process: After transfer, BP continued to fall markedly and he frequently fainted and required assistance with nearly all activities of daily living (ADL). Neuromuscular electrical stimulation (NMES) of the thigh and lower leg was performed five times a week for 30 min. After approximately 3 days of NMES, BP decreased slowly, presyncopic symptoms disappeared, and he could leave bed more frequently and for longer periods. The patient became independent in ADL and was discharged on Day 142. Results at discharge were as follows: SPPB, 11/12; CS5, 13.5; FIM, 114/126. Discussion: Although NMES is not effective for orthostatic hypotension (OH) associated with diabetic autonomic neuropathy (DAN), stabilization of BP early after the introduction of NMES may have been due to its peripheral sympathetic nerve-stimulating effect. Conclusion: The combination of exercise therapy and NMES for OH caused by DAN can alleviate hypotension.

## Introduction

Orthostatic hypotension (OH) is a common yet often overlooked disorder arising from a variety of causes, limiting daily life, and is associated with physical weakness, falls, syncope, cognitive impairment, and risk of death^1^^,^^2)^. Its prevalence is age dependent, ranging from 5% in patients younger than 50 years to 30% in those older than 70 years^3)^. OH is commonly associated with various conditions, including neurodegenerative and autoimmune diseases, diabetes, hypertension, heart failure, and renal failure^2)^.

The treatment of diabetes is important for controlling blood glucose levels and may reduce the occurrence and progression of complications. In addition, a multidisciplinary approach, including pharmacotherapy and exercise therapy, is necessary to address symptoms and complications^4^^,^^5)^. Complications due to microvascular disease in diabetes are most likely to occur in the order of neuropathy, retinopathy, and nephropathy, with the prevalence of OH due to neuropathy estimated at 20%–25%^6^^–^^9)^.

OH is a common complication of diabetic autonomic neuropathy (DAN), and its symptoms are caused by a sudden drop in blood pressure (BP) upon standing up, resulting in dizziness or fainting. DAN causes OH by affecting both the sympathetic and parasympathetic nerves, impairing autonomic nervous system function. Its management is also important because the prevalence of OH increases with the duration of diabetes mellitus, and the complications of OH can reduce the quality of life of patients and even lead to fatal complications^9)^.

Neuromuscular electrical stimulation (NMES) is widely used to prevent muscle weakness after surgery and acute illness^10^^–^^13)^. NMES induces muscle contractions and improves muscle strength and endurance, thereby reducing muscle weakness. Although NMES is generally used to prevent muscle weakness, its efficacy in OH has been reported in patients with spinal cord injury (SCI)^14^^–^^16)^. However, information on the efficacy of NMES for OH complicated by DAN is limited and its effectiveness has not been clearly demonstrated. In the present study, we report our experience with a patient with stable angina pectoris in whom NMES was effective in treating OH due to long-term disuse and DAN.

## Case

The patient was a 75-year-old man with a history of diabetes mellitus since age 70 years, hypertension since age 75 years, hyperchloremia, ischemic heart disease, and OH. He became immobile at home because of loss of appetite and neglect of medication for several days and was promptly taken to a previous physician, where he was admitted on the same day with diabetic ketoacidosis. The patient’s condition was stabilized with intravenous infusion, and physical therapy (PT) was initiated on Day 5. By Day 10, premature ventricular contraction (PVC) increased accompanied by frequent fainting spells, which hindered the patient’s ability to engage in activities. On Day 22, coronary angiography revealed left circumflex branch #15 with 99% stenosis and PVC with pre-exertional syncope. Percutaneous transluminal coronary angioplasty was performed on Day 27. On Day 38, the patient presented with repeated syncope while standing despite the absence of ventricular arrhythmias; based on clinical symptoms, he was diagnosed with OH due to muscle weakness and DAN and was prescribed midodrine. However, syncope episodes persisted despite treatment. On Day 55, the patient was transferred to our hospital to continue PT. Medications included clopidogrel, rosuvastatin, losartan, carvedilol, dapagliflozin, semaglutide, metformin, and midodrine.

**Fig. 1.**
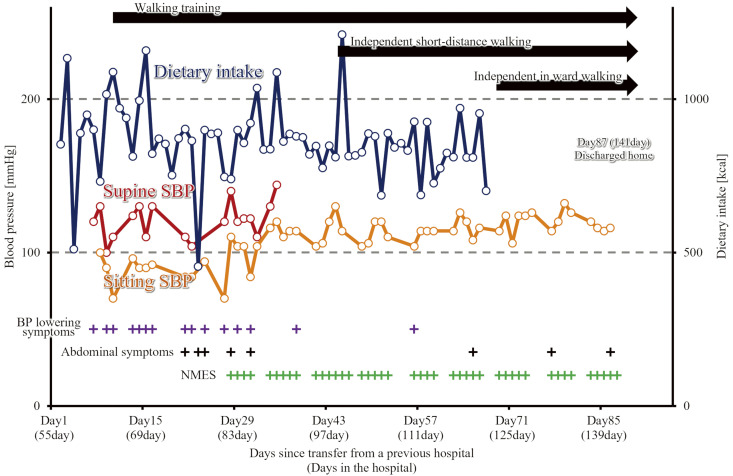
Summary of the patient’s process Dietary and caloric intake did not change, but sitting systolic blood pressure increased and the frequency of hypotensive symptoms decreased markedly after the start of NMES. BP, blood pressure; SBP, systolic blood pressure; NMES, neuromuscular electrical stimulation

The patient continued PT after transfer to our hospital. Table 1 shows the physical function at the time of transfer to and discharge from our hospital. Table 2 shows the laboratory and echocardiographic data, and Figure 1 shows a summary of the patient’s process. BP was persistently low even after sitting exercises, and he had repeated episodes of decreased response or presyncope following walking exercises, accompanied by a decrease in BP. Therefore, based on the recommended intervention^3^^,^^17^^,^^18)^, we performed supine lower limb exercises followed by standing up, graded standing up, and increased fluid intake. However, the patient had a narrow lower leg circumference due to muscle volume loss, making it difficult to apply elastic bandages or stockings. In addition, because of prolonged abdominal symptoms, it was difficult to apply abdominal bandages.

**Table 1.** Physical function assessment

		Admission	Discharge
Height	[cm]	160	
Body weight	[kg]	48.8	49.5
BMI		19.1	19.3
Hand grip	[kg]	–/15.6 (unable to measure due to right wrist pain from overuse)	12.5/13.4
Cuff circumference	[cm]	25.0/25.6	26.2/27.2
SPPB	[points]	1 (0:1:0)	11 (4:4:3)
CS5	[sec]	–	13.5
FIM—total	[points]	66	114
FIM—motor	[points]	35	83
FIM—cognitive	[points]	31	31
BI	[points]	35	90
Standing test (SBP/DBP/HR)	[mmHg/mmHg/bpm]	Supine:130/60/76 Sitting: 90/56/79 Standing: 70/–/79	Supine:140/54/60 Sitting: 116/56/64 Standing: 104/54/71

Hand grip was measured twice in the chair-sitting position with the upper arm drooped and in the middle position of internal and external rotation; the maximum value was used. Cuff circumference was measured three times in the supine position at the maximum circumference (the most distended part of the triceps), and the average value was used. Both hand grip and cuff circumference are listed as right/left.

BMI, body mass index; SPPB, short physical performance battery; CS5, chair stand 5 times; FIM, functional independent measure; BI, Barthel index; SBP, systolic blood pressure; DBP, diastolic blood pressure; HR, heart rate; bpm, beats per minute

**Table 2.** Laboratory and echocardiographic data

	[Unit]	Admission	Discharge
TP	[g/dL]	5.9	6.8
Albumin	[g/dL]	3.4	4.4
Total bilirubin	[mg/dL]	0.3	0.6
ChE	[IU/L]	185	221
AST	[IU/L]	19	51
ALT	[IU/L]	11	40
LDH	[IU/L]	145	176
γ-GTP	[IU/L]	12	25
BUN	[mg/dL]	32.4	30.9
Creatinine	[mg/dL]	1.43	1.3
eGFR	[mL/min/1.73m^2^]	38.0	42.2
WBC	[×10^3^/mm^3^]	5.6	4.0
RBC	[×10^4^/mm^3^]	376	420
Hemoglobin	[g/dL]	11.7	13.1
BNP	[pg/mL]	65.4	
HbA1c	[%]	6.0	6.1
GNRI		86.8	102.2
PNI		40.5	50.6
MNA-SF		2	
LAD	[mm]	32.5	32.6
LVDd	[mm]	40.3	37.6
LVDs	[mm]	26.1	23.8
IVS	[mm]	8.4	9.0
LVPW	[mm]	9.3	9.7
LVEF	[%]	65.2	55.9
E/A		0.60	0.77
DecT	[msec]	190	194
ePAP	[mmHg]	27.0	26.7
IVC	[mm]	8.9	9.6

TP, total protein; ChE, cholinesterase; AST, aspartate transaminase; ALT, alanine transaminase; LDH, lactate dehydrogenase; γ-GTP, γ-glutamyltransferase; BUN, blood urea nitrogen; eGFR, estimated glomerular filtration rate; WBC, white blood cell; RBC, red blood cell; BNP, brain natriuretic peptide; HbA1c, hemoglobin A1c; GNRI, Geriatric Nutritional Risk Index; PNI, Prognostic nutritional index; MNA-SF, Mini Nutritional Assessment Short-Form; LAD, left atrial diameter; LVDd, left ventricular end-diastolic diameter; LVDs, left ventricular end-systolic diameter; IVST, interventricular septum thickness; LVPW, left ventricular posterior wall thickness; LVEF, left ventricular ejection fraction; E/A, early diastolic filling wave/atrial filling wave; DecT, deceleration time; ePAP, estimated pulmonary artery pressure; IVC, inferior vena cava

However, the frequency of BP drop and presyncope did not decrease, and on Day 83 (the 28th day after transfer), NMES was started, and the diet was changed to increase the amount of food and salt intake (noodles increased in frequency according to the patient’s preference). NMES (Sorius SOL-1; Minato Medical Science Co., Ltd., Osaka, Japan) was performed 5 times a week for 30 min at an intensity of visible muscle contraction in the thigh and lower legs.

Approximately 3 days after the start of NMES, although dietary and caloric intake did not change, the BP drop on awakening became slower, and BP lowering symptoms and the frequency of presyncope decreased (Fig. 1). This enabled the patient to increase the time away from bed and actively perform strength training in the anti-gravity position and walking exercises, which gradually enabled the increased activities of daily living (ADL). During hospitalization, NMES was continued, and as physical function improved, the patient became independent in ADLs. However, since the patient had difficulty becoming independent in instrumental ADL, such as going out and shopping, the patient was discharged on Day 142 (the 87th day after transfer) with the help of nursing welfare services. The patient had no syncope or falls for approximately 1 month after discharge.

## Discussion

In the present case, an elderly male patient had OH associated with DAN and showed an inadequate response to conventional therapies. Because of the presence of underlying cardiac disease, conventional recommendations such as increases in salt intake and changes in guideline-directed medical therapy were not consented to, and commonly recommended measures to deal with OH^3^^,^^17^^–^^20)^, such as physical counter maneuver methods and elastic stockings, could not be applied because of the marked decline in physical function.

However, with the introduction of NMES, the frequency of hypotensive symptoms and presyncope decreased, and physical function improved. Non-pharmacological interventions for OH, including electrical stimulation, have not shown clear efficacy^21)^. Furthermore, the effectiveness of NMES in the management of OH associated with DAN has not yet been reported in previous studies. In other words, there are likely large individual differences in intervention efficacy.

However, the preventive effect of NMES on OH by lowering BP during orthostasis has been observed in patients with SCI^14^^–^^16^^,^^22^^–^^27)^ and stroke^28)^. Regarding the preventive effect of NMES on hypotension in patients with SCI, the BP response to electrical stimulation was independent of the stimulation site and was not due to an increase in venous return secondary to muscle pumping or a hypertonic reflex response to isometric muscle contraction. Instead, the (unpleasant or noxious) stimulus itself may cause an autonomic dysreflexia type of sympathetic response^14)^.

Diabetic neuropathy, considered a factor in OH in this case, is believed to occur in parallel and simultaneously with changes in neurons, Schwann cells, and axons, as diabetes causes abnormalities in the neurotrophic and microvascular blood supply to the ganglia^6^^,^^8)^. Another factor, disuse due to rest and low activity, is known to deteriorate both the quantity and quality of skeletal muscle and decrease skeletal muscle recruitment^29)^. The NMES in the present case was adjusted to an intensity that was accompanied by muscle contraction and joint movement without discomfort, that is, unlikely to cause retrograde inhibition (NMES ≤10% maximal voluntary contraction as a guide)^30)^. The beneficial effect on OH, despite the lack of strong stimulation as in previous studies, may be due to the increased recruitment of skeletal muscle by NMES^10^^,^^31^^,^^32)^ and the improved nerve conduction resulting from better blood flow to the peripheral nerves^33)^. The results suggest that NMES may be an effective intervention for OH associated with DAN in the future.

Decreased physical activity and immobilization are also important factors that cause loss of muscle mass. Immobilization-induced muscle atrophy is accompanied by a decrease in extracellular Ca_2_^+^ influx via Piezo1, which promotes muscle atrophy by increasing KLF15 expression^34)^. Furthermore, in patients with diabetes mellitus, inhibition of skeletal muscle Ca_2_^+^ uptake capacity is more likely to occur during rest, and exercise also inhibits Ca_2_^+^ uptake capacity^35)^. Therefore, patients with diabetes mellitus are prone to early muscle dysfunction under resting conditions, and interventions such as NMES are important to prevent muscle dysfunction.

The results of this case suggest that NMES is a promising therapy for managing OH associated with DAN. NMES is effective in ameliorating not only the symptoms of OH due to muscle weakness as conventionally thought but also through neurophysiological mechanisms.

However, the report describes only one case, and the neurophysiological mechanism of NMES is unclear. Further studies are needed to elucidate the mechanism that prevents hypotension and the effect of NMES. In addition, the effect of OH due to the vasodilatory response associated with the α-receptor stimulating effect of αβ-blockers and the reduction of OH symptoms over time cannot be ruled out. In addition, the optimization of NMES and treatment protocols needs to be studied. We hope that future studies will clarify the potential of NMES as an effective treatment for OH.

## Conclusion

The combination of exercise therapy and NMES reduced hypotensive symptoms in patients with stable angina pectoris complicated by OH secondary to DAN. Therefore, NMES may be a promising therapy for the management of OH associated with DAN.

## Ethical considerations

The study was conducted in accordance with the principles outlined in the Declaration of Helsinki and was approved by the ethics committee of Sanseikai Kitano Hospital (approval number: 24-1). Informed consent was obtained from the patient for publication of this report. The authors confirm that the manuscript contains no identifying information concerning the participants and that the information has been fully anonymized. Furthermore, the authors affirm that all mandatory health and safety procedures were followed during the experimental work reported in this paper.

## Acknowledgments

We would like to thank Editage for assistance with English language editing. The results of the study are presented clearly, honestly, and without fabrication, falsification, or inappropriate data manipulation, and the results of the present study do not constitute an endorsement by the Journal of Physical Therapy Research.

## Funding

This study received no funding. This study was conducted at the Department of Rehabilitation, Sanseikai Kitano Hospital, Sapporo, Japan.

## Availability of data and materials

The dataset used in this study is available from the corresponding author upon request.

## Consent for publication

Not applicable.

## Conflicts of Interest

The authors declare that they have no conflicts of interest.
